# Autoantibodies to Oxidatively Modified Peptide: Potential Clinical Application in Coronary Artery Disease

**DOI:** 10.3390/diagnostics12102269

**Published:** 2022-09-20

**Authors:** I-Jung Tsai, Wen-Chi Shen, Jia-Zhen Wu, Yu-Sheng Chang, Ching-Yu Lin

**Affiliations:** 1 Ph.D. Program in Medical Biotechnology, College of Medical Science and Technology, Taipei Medical University, Taipei 11031, Taiwan; 2 Institute of Biotechnology, National Tsing Hua University, Hsinchu 30013, Taiwan; 3 School of Medical Laboratory Science and Biotechnology, College of Medical Science and Technology, Taipei Medical University, Taipei 11031, Taiwan; 4 Division of Allergy, Immunology and Rheumatology, Department of Internal Medicine, Shuang Ho Hospital, New Taipei City 23561, Taiwan; 5 Division of Allergy, Immunology and Rheumatology, Department of Internal Medicine, School of Medicine, College of Medicine, Taipei Medical University, Taipei 11031, Taiwan

**Keywords:** plasma, 4-hydroxynonenal, machine learning, oxidative stress

## Abstract

Coronary artery disease (CAD) is a global health issue. Lipid peroxidation produces various by-products that associate with CAD, such as 4-hydroxynonenal (HNE) and malondialdehyde (MDA). The autoantibodies against HNE and MDA-modified peptides may be useful in the diagnosis of CAD. This study included 41 healthy controls (HCs) and 159 CAD patients with stenosis rates of <30%, 30–70%, and >70%. The plasma level of autoantibodies against four different unmodified and HNE-modified peptides were measured in this study, including CFAH^1211–1230^, HPT^78–108^, IGKC^2–19^, and THRB^328–345^. Furthermore, feature ranking, feature selection, and machine learning models have been utilized to exploit the diagnostic performance. Also, we combined autoantibodies against MDA and HNE-modified peptides to improve the models’ performance. The eXtreme Gradient Boosting (XGBoost) model received a sensitivity of 78.6% and a specificity of 90.4%. Our study demonstrated the combination of autoantibodies against oxidative modification may improve the model performance.

## 1. Introduction

Coronary artery disease (CAD) is a global health care issue which affected nearly 1.72% of individuals worldwide [1]. The progression of CAD consists of three stages: fatty streak, plaque progression, and disruption. Starting from inflammatory cells recruitment and lipid accumulation, the inflammation may lead to plaque formation and eventually cause atherosclerosis [2]. Inflammation occurs with the accumulation of free radicals, which may accelerate the process of CAD progression and create a positive feedback loop to form additional free radicals [3]. Inflammation has been considered as the major factor of CAD progression. Hence, the diagnosis of CAD in the clinic focuses on the measurement of inflammation markers, such as C-reactive protein (CRP), cytokines, and adhesion molecules [4].

In addition to inflammation, lipid peroxidation can also generate free radicals; it has been considered as a process of oxidants’ attack on the lipids containing carbon–carbon double bonds [5]. Lipid peroxidation produces several by-products such as malondialdehyde (MDA), propenal (acrolein), hexanal, and 4-hydroxynonenal (4-HNE), which are associated with chronic diseases [5,6,7,8,9]. These by-products may modify macromolecules, and cause damage to the molecular functions of the macromolecules [6]. Furthermore, since MDA and 4-HNE are extremely toxic by-products derived from lipid peroxidation [10], numerous studies have reported the pathological processes with the participation of MDA and 4-HNE [5]. While MDA is the most abundant by-product in lipid peroxidation, 4-HNE shows the highest biological activity during the oxidation.

Although MDA has been considered as a reliable and popular biomarker for evaluating oxidative stress, an improvement in identifying free form MDA and total MDA is required [5,11]. A common detection method for MDA, a thiobarbituric acid (TBA) assay, exhibited non-specificity, poor reproducibility, lack of the recovery test results, and instability of MDA [12]. Aside from MDA, 4-HNE has recently been brought to researchers’ attention due to several advantages such as high stability. Not only does 4-HNE produce a large amount in tissues, but it also exhibits higher stability compared to MDA [13,14]. The formation of 4-HNE is attributed to the decomposition of the primary products of lipid peroxidation; once lipid hydroperoxides transform into peroxyl and alkoxyl (LO) radicals, they may form secondary products such as 4-HNE [15]. Part of the 4-HNE compounds enter the process of biotransformation, whereas part of the compounds form 4-HNE adducts by conjugating with various cellular components such as protein and DNA. Both 4-HNE modified adducts lead to genotoxicity and protein dysfunction [14]. The 4-HNE-modified proteins disrupt the process of protein degradation and eventually cause metabolism diseases, such as atherosclerosis and rheumatological diseases [16]. In addition, the protein modifications of MDA and 4-HNE are considered oxidation-specific epitopes (OSE) and recognized by autoantibodies [17,18]. OSE also presented on oxidized LDL (OxLDL), which has been associated with an increased risk of cardiovascular disease [19]. Circulating autoantibodies against OSE are involved in the initiation and the formation of atherosclerosis. Furthermore, OSE on lipoprotein can be measured as a biomarker in CAD [18]. 

Previously, we discovered four novel HNE-modified peptides in serum derived from patients with RA [20]. The 4-HNE-modified positions on the peptides were underlined: ^1211^-SHTLRTTCWDGKLEYPTCAK-^1230^ (complement factor H, CFAH^1211–1230^), ^78^-AVGDKLPECEADDGCPKPPEIAHGYVEHSVR-^108^ (haptoglobin, HPT^78–108^), ^2^-TVAAPSVFIFPPSDEQLK-^19^ (immunoglobulin kappa constant, IGKC^2–19^), and ^328^-TFGSGEADCGLRPLFEKK-^345^ (prothrombin, THRB^328–345^). Crowson et al. suggested that patients with RA have a twofold increased risk of developing CAD [21]. Moreover, it is one of the leading causes of death in patients with RA in Taiwan [22]. Hence, we speculated the autoantibodies we discovered in patients with RA may be useful in the diagnosis of CAD. In this study, we measured HNE-modified adducts and autoantibodies against unmodified and HNE-modified peptides in HCs and CAD patients with stenosis rates of <30%, 30–70%, and >70%. Furthermore, we incorporated feature ranking and selection techniques to optimize our machine learning models. Finally, the models were evaluated by accuracy, precision, f1 score, sensitivity, specificity, and area under the receiving operating characteristic curve (AUC).

## 2. Materials and Methods

### 2.1. Patients Sample

**Figure 1:**
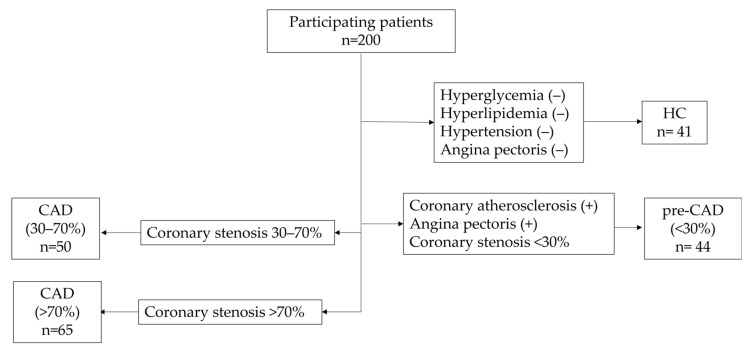
The participants were grouped into HC, pre-CAD (<30%), CAD (30–70%), and CAD (>70%).

Plasma samples from 30 patients with RA (12 female and 18 male patients) and 30 patients with RA-CAD (11 female and 19 male patients) were obtained from the Division of Allergy, Immunology, and Rheumatology, Department of Internal Medicine and the Department of Laboratory Medicine, Shuang-Ho Hospital (NTPC, New Taipei City, Taiwan). Plasma samples from 159 patients with CAD (51 female and 108 male patients) and 41 healthy controls (HCs) were obtained from Cardiovascular Center of the Lo-Hsu Medical Foundation Luodong Poh-Ai Hospital. Patients defined as having pre-coronary artery disease were recorded with coronary atherosclerosis or angina pectoris, and were found to have coronary artery stenosis rate <30%. In the groups of patients with coronary artery disease, patients were separated into the group of coronary stenosis rate of 30–70% and the group of coronary stenosis rate of >70%. CAD patients who also were diagnosed as RA were excluded (Figure 1). As for comorbidity, patients were considered as having hyperglycemia with one of following criteria: total cholesterol ≥200 mg/dL, LDL-C ≥ 130 mg/dL, TG ≥ 200 mg/dL, and HDL-C < 40 mg/dL, or receiving lipid lowering drug prescription. Patients were considered as having hypertension with one of following criteria: systolic blood pressure ≥140, diastolic blood pressure ≥90, or receiving an antihypertensive drug prescription. Patients were considered as having diabetes with one of following criteria: diagnosis with diabetes or receiving a hypoglycemia drug prescription. Healthy controls (HCs) were excluded if they suffered from hyperglycemia, hyperlipidemia, hypertension, or angina pectoris. Ten mL of blood samples were collected from patients and HCs. After the blood samples were centrifuged 3000 rpm for 10 min, the plasma was stored at −80 °C until analyzed. This study was approved by the institutional review board of the study hospital, and all volunteers provided informed consent before participating. Patient samples were randomly selected. The demographic characteristics of patients are summarized in Table 1. The Taipei Medical University-Joint Institutional Review Board and the Institutional Review of Cathay General Hospital approved the study protocol (N201512049 (3 February 2017), CGP-LP106006 (15 June 2017)).

### 2.2. Detection of Plasma HNE Adducts

HNE-modified BSA (A7906, Sigma, Neustadt, Germany) standards (100 µL) or diluted samples (10 µg/mL) were added into a 96-well plate (Thermo Fisher, Waltham, MA, USA) and incubated at 37 °C for 2 h. For blocking, 3% of a BSA solution was added and incubated at 37 °C for 1 h after the plates were washed with PBS containing 0.05% Tween 20 (PBST). The plates were washed with PBST, and a rabbit anti-HNE antibody (ab46545, Abcam, Cambridge, MA, USA) was added. After incubating for 2 h at room temperature, the plates were washed with PBST. A mouse anti-rabbit antibody conjugated with horseradish peroxidase (HRP) was added into plates and incubated for 1 h. We washed the plates with PBST and detected the HRP with SureBlue Reserve™ TMB (Kirkegard and Perry Laboratories, Gaithersburg, MD, USA) for 30 min. The color reaction was stopped with 1N HCl, and the absorbance was measured at 450/620 nm. The concentration of HNE-protein adducts in plasma was calculated according to a standard HNE-modified BSA curve.

### 2.3. Detection of Plasma Autoantibodies against Unmodified and HNE Modified Peptide

The BSA and four peptides with 1mg/mL were modified with 4-Hydroxynonenal (Sigma, Neustadt, Germany). The BSA or peptides (10 µg/mL) were added into a 96-well plate and incubated at 37 °C for 2 h. For blocking, after washing with PBST, 3% of a BSA solution was added and incubated at 37 °C for 1 h. The plates were washed with PBST and the 100-fold diluted plasma samples were added. The plates were incubated at room temperature for 2 h. After the plates were washed, rabbit anti-human IgG-HRP (A80-118P, 1:30,000, BETHYL) or rabbit anti-human IgM-HRP (A0420, 1:10,000, Sigma) were diluted and added into plates. The plates were incubated at room temperature for 1 h. After that, the plates were washed completely and added with the SureBlue ReserveTM Peroxidase Substrate (Kirkegard & Perry Laboratories, Gaithersburg, MD, USA). The plates were incubated at room temperature for 15 min. The color reaction was stopped by adding 1N HCl, and the absorbance was measured at 450/620 nm. All ELISA experiments were conducted following the ELISA Guidebook. The quality controls samples were prepared with two replicates in each plate to calculate the percent coefficient of variation (CV%) across wells and plates. An experiment was repeated if the CV% was calculated above 20%.

### 2.4. Statistical Analysis and Machine Learning

The significance of IgG and IgM autoantibodies between HCs and RA, RA-CAD, or CAD patients were determined by Student’s *t*-test. The Student’s *t*-test was calculated by GraphPad Prism (v.8.0; GraphPad software, San Diego, CA, USA). The significance level of all statistical tests was set to *p* < 0.05. The feature ranking was conducted by WEKA (vers.3.8.5). The models we built in this study were based on eXtreme Gradient Boosting (XGBoost) and the Light Gradient Boosting Machine (GBM) with 5-fold cross validation with scikit-learn (vers.0.21.3). A confusion matrix was applied in this study to calculate the accuracy, precision, sensitivity, specificity, and f1 score. The value of AUC was calculated with scikit-learn (vers.0.21.3). The comparison of models was evaluated by an ANOVA test.

## 3. Results

### 3.1. Measurement of Autoantibodies against HNE Modified BSA

Plasma samples were subjected to ELISAs for measuring IgG and IgM autoantibodies against unmodified and HNE-modified BSA. Plasma levels of IgG and IgM against HNE-modified BSA were found to be increased in patients with RA and RA-CAD (Supplementary Figure S1A,B). Plasma levels of IgM against BSA were found significantly different between HC and RA (Supplementary Figure S1B).

### 3.2. Measurement of Autoantibodies against HNE-Modified Peptides and HNE Adducts

Plasma samples were analyzed with ELISA to detect IgG and IgM autoantibodies against unmodified and HNE-modified peptides (Supplementary Figure S2). Plasma levels of IgG against the CFAH^1211–1230^ unmodified peptide and HNE-modified peptide in CAD patients with a stenosis rate >70% were notably higher than HCs (*p* = 0.008, *p* = 0.0002). Plasma levels of IgM against the CFAH^1211–1230^ unmodified peptide and HNE-modified peptide in CAD patients with a stenosis rate >70% were significantly lower than HCs (*p* < 0.0001, *p* < 0.0001) and CAD patients with a stenosis rate <30% (*p* = 0.0092, *p* = 0.0211). Plasma levels of IgG against the HPT^78–108^ unmodified peptide and HNE-modified peptide in CAD patients with a stenosis rate >70% were significantly decreased compared to HCs (*p* = 0.0002, *p* = 0.0001). Plasma levels of IgM against the HPT^78–108^ unmodified peptide and HNE modified peptide in CAD patients with a stenosis rate >70% were notably lower than HCs (*p* < 0.0001, *p* < 0.0001). Plasma levels of IgG against the IGKC^2–19^ unmodified peptide in CAD patients with a stenosis rate >70% were significantly lower than HCs (*p* < 0.0001). In contrast, plasma levels of IgG against the IGKC^2–19^ HNE-modified peptide in CAD patients with stenosis rates of <30%, 30–70%, and >70% were decreased compared to HCs (*p* = 0.02, *p* = 0.001, *p* = 0.0002). Plasma levels of IgM against the IGKC^2–19^ unmodified peptide in CAD patients with a stenosis rate >70% were lower than HCs (*p* < 0.0001) and CAD patients with a stenosis rate <30% (*p* = 0.0056). Plasma levels of IgM against IGKC HNE-modified peptide in CAD patients with stenosis rates of <30%, 30–70%, and >70% were lower than HCs (*p* = 0.0001, *p* = 0.0005, *p* < 0.0001). Plasma levels of IgG against the THRB^328–345^ HNE-modified peptide in CAD patients with a stenosis rate >70% were higher than HCs (*p* < 0.0001) and CAD patients with stenosis rate <30% (*p* = 0.0039). Plasma levels of IgM against the THRB^328–345^ unmodified and HNE-modified peptide in CAD patients with a stenosis rate >70% were significantly higher than HCs (*p* = 0.0003, *p* = 0.0002) and CAD patients with stenosis rate <30% (*p* = 0.00498, *p* = 0.00486). Furthermore, HNE-modified protein adducts in patients with CAD were higher than HCs (Table 1).

### 3.3. Optimization of Machine Learning Algorithms with Autoantibodies against HNE Modified Peptides

To increase the performance of LightGBM and XGBoost, we firstly performed feature ranking with InfoGain + Ranker to list the features from the most important to less important in HCs against patients with stenosis rates of <30%, 30–70%, and >70% (Table 2). The autoantibody, IgG anti-IGKC^2–19^ HNE, was ranked as the top feature in each analysis. Next, we performed forward selection to further optimize LightGBM and XGBoost. In the LightGBM model, the features that were selected included IgG anti-IGKC^2–19^ HNE, IgM anti-CFAH^1211–1230^, IgG anti-CFAH^1211–1230^, IgM anti-CFAH^1211–1230^ HNE, IgG anti-CFAH^1211–1230^ HNE, IgM anti-HPT^78–108^, IgM anti-HPT^78–108^ HNE, IgG anti-HPT^78–108^ HNE, IgM anti-IGKC^2–19^, IgG anti-IGKC^2–19^, IgM anti-IGKC^2–19^ HNE, IgM anti-THRB^328–345^, IgG anti-THRB^328–345^, IgM anti-THRB^328–345^ HNE, and IgG anti-THRB^328–345^ HNE. In differentiating HCs and CAD patients with a stenosis rate of <30%, the model received an accuracy of 77%, a precision of 73%, a f1 score of 72.6%, a sensitivity of 78.2%, a specificity of 76.4%, and an AUC value of 0.832. In discriminating HCs and CAD patients with a stenosis rate of 30–70%, the model received an accuracy of 72.7%, a precision of 72.5%, a f1 score of 69.1%, a sensitivity of 71.5%, a specificity of 75.8%, and an AUC value of 0.816. As for HCs and CAD patients with stenosis rates of >70%, the model received an accuracy of 73%, a precision of 61%, a f1 score of 57.8%, a sensitivity of 62%, a specificity of 79.6%, and an AUC value of 0.819. In the XGBoost model, the features that were selected included IgG anti-IGKC^2–19^ HNE, IgG anti-CFAH, IgG anti-HPT^78–108^, IgM anti-HPT^78–108^ HNE, IgM anti-IGKC^2–19^, IgG anti-IGKC^2–19^, and IgM anti-IGKC^2–19^ HNE (Table 3). In discriminating HCs and CAD patients with stenosis rates of <30%, the model received an accuracy of 77.7%, a precision of 74.5%, a f1 score of 71.4%, a sensitivity of 74.2%, a specificity of 79.2%, and an AUC value of 0.845. As for HCs and CAD patients with stenosis rates of 30–70%, the model received an accuracy of 75.3%, a precision of 71.7%, a f1 score of 71.4%, a sensitivity of 75.6%, a specificity of 75.4%, and an AUC value of 0.825. In differentiating HCs and CAD patients with stenosis rates of >70%, the model received an accuracy of 77.3%, a precision of 66.9%, a f1 score of 62.8%, a sensitivity of 64.5%, a specificity of 83.2%, and an AUC value of 0.856 (Table 3).

### 3.4. Optimization of Machine Learning Algorithms with Autoantibodies against HNE and MDA-Modified Peptides

**Figure 2:**
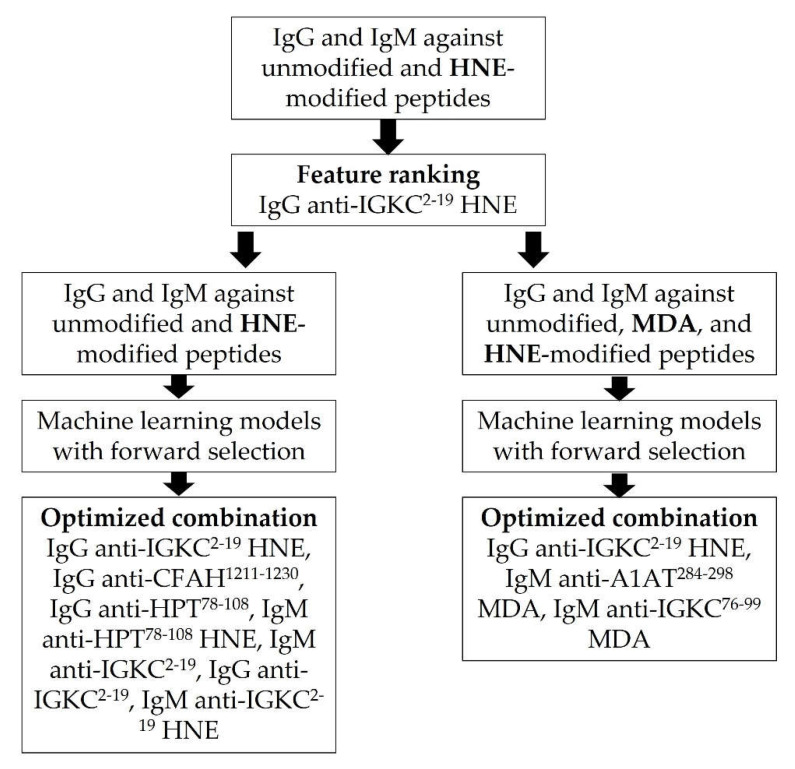
The flowchart of the developing oxidative model.

In our previous study, we had reported that the autoantibodies against four different MDA modified peptides may serve as biomarkers in diagnosing patients with CAD [23]. The MDA-modified positions were underlined: ^76^-ADYEKHKVYACEVTHQGLSSPVTK-^99^ (IGKC^76–99^), ^284^-LQHLENELTHDIITK-^298^ (alpha-1-antitrypsin, A1AT^284–298^), ^824^-VSVQLEASPAFLAVPVEK-^841^ (alpha-2-macroglobulin, A2M^824–841^,), and ^4022^-WNFYYSPQSSPDKKLTIFK-^4040^ (apolipoprotein B-100, ApoB100^4022–4040^). The IgG and IgM autoantibodies against unmodified, MDA, and HNE-modified peptides were summarized in Table 4. The autoantibody, IgG anti-IGKC^1–18^ HNE, was selected as the first feature. We then performed forward selection with autoantibodies against HNE and MDA-modified peptides (Table 5, Figure 2). In the LightGBM model, the features selected included IgG anti-IGKC^1–18^ HNE, IgM anti-A1AT MDA, IgM anti-IGKC^1–18^ MDA, IgG anti-A2M MDA, IgG anti-A1AT MDA, and IgM anti-CFAH HNE. In differentiating HCs and CAD patients with a stenosis rate of <30%, the model received an accuracy of 75.7%, a precision of 74.8%, a f1 score of 72%, a sensitivity of 75.1%, a specificity of 77.2%, and an AUC value of 0.848. As for HCs and CAD patients with a stenosis rate of 30–70%, the model received an accuracy of 76.1%, a precision of 72.2%, a f1 score of 71.3%, a sensitivity of 75.7%, a specificity of 76.6%, and an AUC of 0.845. In discriminating HCs and CAD patients with a stenosis rate of >70%, the model received an accuracy of 82.7%, a precision of 74.5%, a f1 score of 71.9%, a sensitivity of 75.2%, a specificity of 86.4%, and an AUC value of 0.904. In the XGBoost model, the features selected included IgG anti-IGKC^1–18^ HNE, IgM anti-A1AT MDA, and IgM anti-IGKC^1–18^ MDA. In the discrimination of HCs and CAD patients with a stenosis rate of <30%, the model received an accuracy of 78.2%, a precision of 76.8%, a f1 score of 75%, a sensitivity of 78.4%, a specificity of 78.8%, and an AUC value of 0.847. As for HCs and CAD patients with a stenosis rate of 30–70%, the model received an accuracy of 78.6%, a precision of 76.6%, a f1 score of 74.2%, a sensitivity of 77.1%, a specificity of 79.9%, and an AUC value of 0.881. In the differentiation of HCs and CAD patients with a stenosis rate of >70%, the model received an accuracy of 86.1%, a precision of 81.7%, a f1 score of 77.2%, a sensitivity of 78.6%, a specificity of 90.4%, and an AUC value of 0.935. Statistical tests were performed to validate the improvement.

## 4. Discussion

In this study, we examined the diagnostic performance of four HNE-modified peptides in CAD that had been previously reported in patients with RA [20]. Autoantibodies found in RA have been related to cardiovascular events [24]. Thus, we speculated that the autoantibodies we discovered previously may be useful in the diagnosis of CAD. We firstly examined the IgG and IgM autoantibodies against BSA and HNE-modified BSA (Supplementary Figure S1). The results indicated the diagnostic potential of IgG and IgM autoantibodies against HNE-modified adducts. Hence, we measured the IgG and IgM autoantibodies against peptides, including CFAH, HPT, IGKC^2–19^, THRB, and HNE-modified peptides. We found that IgG and IgM autoantibodies against CFAH, HPT, IGKC^2–19^, THRB, and HNE-modified peptides decreased in CAD patients.

The proteins were discovered to be associated with the development of CAD. For instance, CFAH has been found in early human coronary atherosclerotic lesions [25]. Lee et al. suggested that HPT may be elevated in the plasma derived from CAD patients [26]. Although no study has reported the elevation of IGKC in CAD patients, MDA-modified IgG was found to be significantly elevated in CAD patients [27]. The elevation of THRB fragments was reported in patients with peripheral artery disease (PAD), which is also a cardiovascular risk factor [28,29]. Increasing the level of 4-HNE and above-mentioned proteins in CAD patients may accelerate the increment of oxidized proteins, which play a crucial role in the formation of atherosclerosis. Therefore, the 4-HNE modified peptides may also trigger inflammation and strengthen the development of atherosclerosis. Studies indicated that autoantibodies may be associated with the severity of CAD [30,31]. The decreased level of autoantibodies against 4-HNE modified peptides possibly indicates the clearance of 4-HNE or immune dysregulation [32,33]. However, the role of autoantibodies against MDA and 4-HNE modified peptides in the development of CAD requires further investigation.

To date, no other study has investigated autoantibodies against HNE-modified peptides in patients with cardiovascular disease. However, HNE increment has been associated with oxidative stress and vascular disease [34,35]. In addition, the formation of HNE-modified adducts may induce the generation of autoantibodies [36]. Hence, we speculated that the decreasing level of IgG and IgM against HNE-modified peptides may result from the immune dysfunction of patients with CAD [37].

To further explore the diagnostic ability, we conducted feature selection (feature ranking + forward selection) and machine learning models. IgG anti-IGKC^2–19^ HNE was ranked as the most important feature during each ranking (Table 2). Thus, the following forward selection was initiated with IgG anti-IGKC^2–19^ HNE. Various types of machine learning algorithms have been applied into medical studies. Examples include decision tree, support vector machine, random forest, and neural network [38]. Recently, two advanced tree-based models, Light Gradient Boosting Machine (lightGBM) and eXtreme Gradient Boosting (XGBoost), have been popular in other studies. Wang et al. built their miRNA classifier with LightGBM and identified hsa-mir-139 as an important feature for breast cancer diagnosis [39]. Joo et al. analyzed the cohort data from Korean National Health and developed various machine learning prediction models to estimate 2-year and 10-year risks of cardiovascular disease (CVD). LightGBM owned the highest AUC value in estimating 10 year follow-ups without medication features [40]. Al’Aref et al. incorporated clinical features and coronary artery calcium scores (CACs) with an XGBoost model to estimate the pretest of obstructive CAD on coronary computed tomography angiography (CCTA). They received the best performance with an AUC of 0.866 [41]. Kim et al. enrolled 1312 patients with obstructive CAD on coronary angiography. They built a model with the XGBoost algorithm and received an AUC of 0.820 as the best model in their experiments [42].

In our study, we performed forward feature selection with LightGBM and XGBoost. We received the highest performance with features including IgG anti-IGKC^2–19^ HNE, IgM anti-A1AT^284–298^ MDA, and IgM anti-IGKC^76–99^ MDA. The XGBoost model received a sensitivity of 0.786 and a specificity of 0.904. Many biomarkers have been utilized in the clinic. For instance, the C-reactive protein (CRP) has been considered as a useful biomarker to predict cardiac death, AMI, and heart failure [43]. In addition, high sensitivity-CRP (hs-CRP) was found mildly elevated (up to 15 mg/L) in suspected ACS patients, which may be a meaningful prognostic marker in the clinic [44]. Furthermore, inflammation markers were studied in CAD. For example, the elevation of interleukin-6 (IL-6) was found in induced myocardial infarction [45]. Cyclophilin A was found elevated significantly in CAD patients with type 2 diabetes. It served as a high specificity biomarker for diagnosis of CAD patients with type 2 diabetes [46]. Furthermore, other researchers focus on circulating protein and RNA [47]. Together, a multiplex biomarker panel may improve the diagnosis with a comprehensive analysis [48]. In this study, we demonstrated a combination of autoantibodies against two types of oxidative modified peptides (MDA and HNE). The XGBoost model was improved the most after we incorporated autoantibodies against MDA and HNE-modified peptides together (Table 5). Our future work may incorporate other oxidative stress related markers to comprehend our oxidative model.

An in vitro diagnostic multivariate index assay (IVDMIA) combines multiple values with an algorithm to reach an improved accuracy compared to a single biomarker [49]. It has been applied to improve the diagnosis of ovarian cancer [50]. In addition, autoantibodies have been considered as valid biomarkers for various diseases such as cancer [51], RA [52], neurodegenerative disease [53], and CAD [54]. Together, these studies indicate the potential of improving diagnostic ability with an IVDMIA combined with an immunoassay in the clinic. However, several limitations should be noted. The machine learning models had been improved after we incorporated multiple autoantibodies against oxidatively modified peptides. Nevertheless, a larger sample size is required for further validation. Furthermore, other biomarkers from the clinic that may improve the diagnosis should be included, such as hs-CRP.

## 5. Conclusions

In this study, we firstly reported the plasma level of autoantibodies IgG and IgM against CFAH^1211–1230^, HPT^78–108^, IGKC^2–19^, and THRB^328–345^ and their HNE-modified peptides. In addition, we incorporated machine learning models to exploit the potential of their diagnostic performance. Moreover, we included autoantibodies IgG and IgM against MDA-modified peptides to further improve the performance of the models. Our study provided a demonstration that combining autoantibodies against two types of oxidative modification may improve the model performance.

## Figures and Tables

**Table 1:** Comparison of clinical characteristics between HC and CAD patients.

Variables	Shuang-Ho Hospital			Luodong Poh-Ai Hospital		
					Stenosis Rate of Patients	
	RA (*n* = 30)	RA-CAD (*n* = 30)	HC (*n* = 41)	<30% (*n* = 44)	30–70% (*n* = 50)	>70% (*n* = 65)
Age (year)	52.26 ± 4.27	53.65 ± 9.19	38.41 ± 10.42	62.72 ± 10.32	63.57 ± 9.55	62.79 ± 9.27
Male	12	26	26	29	23	56 *
Drinker	-	11	11	5	12	10
Used to smoke	-	-	1	15 *	10 *	19
Current smoker	-	-	17	4	11	36 *
Diabetes	-	-	-	13	15	30
Hypertension	-	-		30	39	56
HNE-protein adducts	-	-	1.010 ± 0.088	1.044 ± 0.097	1.054 ± 0.115 *	1.120 ± 0.112 **

* means *p* < 0.05, ** means *p* < 0.0001.

**Table 2:** Feature ranking results among HCs versus CAD patients with stenosis rates of <30%, 30–70% and >70%.

HC vs. <30%		HC vs. 30–70%		HC vs. >70%	
Score	Attributes	Score	Attributes	Score	Attributes
**0.525**	**IgG anti-IGKC HNE**	**0.356**	**IgG anti-IGKC HNE**	**0.288**	**IgG anti-IGKC HNE**
0.27	IgM anti-IGKC HNE	0.275	IgM anti-IGKC HNE	0.278	IgM anti-IGKC HNE
0.209	IgG anti-THRB	0.178	IgM anti-THRB HNE	0.172	IgM anti-THRB HNE
0.167	IgG anti-THRB HNE	0.178	IgG anti-THRB	0.164	IgM anti-THRB
0.14	IgM anti-THRB HNE	0.153	IgG anti-THRB HNE	0.156	IgM anti-HPT
0.127	IgM anti-HPT	0.128	IgM anti-THRB	0.143	IgM anti-CFAH HNE
		0.126	IgM anti-HPT	0.128	IgM anti-CFAH
		0.126	IgM anti-HPT HNE	0.123	IgM anti-HPT HNE
				0.116	IgG anti-THRB
				0.109	IgG anti-HPT
				0.109	IgG anti-THRB HNE
				0.104	IgG anti-CFAH HNE

**Table 3:** The machine learning models incorporated autoantibodies against unmodified and HNE-modified peptides.

	**Groups**	**Accuracy (95%CI)**	**Precision (95%CI)**	**f1 Score (95%CI)**	**Sensitivity (95%CI)**	**Specificity (95%CI)**	**AUC (95%CI)**
LGBM	IgG anti-IGKC HNE, IgM anti-CFAH, IgG anti-CFAH, IgM anti-CFAH HNE, IgG anti-CFAH HNE, IgM anti-HPT, IgM anti-HPT HNE, IgG anti-HPT HNE, IgM anti-IGKC, IgG anti-IGKC, IgM anti-IGKC HNE, IgM anti-THRB, IgG anti-THRB, IgM anti-THRB HNE, IgG anti-THRB HNE						
	HC vs. <30%	77% (64.6–89.4%)	73% (49.5–96.5%)	72.6% (53–92.2%)	78.2% (54.7–101.6%)	76.4% (57.5–95.2%)	0.832 (0.657–1.008)
	HC vs. 30–70%	72.7% (59.2–86.2%)	72.5% (52.5–92.4%)	69.1% (52.9–85.3%)	71.5% (49.6–93.4%)	75.8% (57–94.6%)	0.816 (0.665–0.967)
	HC vs. >70%	73% (59.1–87%)	61% (32.5–89.6%)	57.8% (34.6–81.1%)	62% (33.5–90.5%)	79.6% (64.1–95.2%)	0.819 (0.66–0.978)
	**Groups**	**Accuracy (95%CI)**	**Precision (95%CI)**	**f1 Score (95%CI)**	**Sensitivity (95%CI)**	**Specificity (95%CI)**	**AUC (95%CI)**
XGB	IgG anti-IGKC HNE, IgG anti-CFAH, IgG anti-HPT, IgM anti-HPT HNE, IgM anti-IGKC, IgG anti-IGKC, IgM anti-IGKC HNE						
	HC vs. <30%	77.2% (63.6–90.8%)	74.5% (51.9–97.2%)	71.4% (51.1–91.7%)	74.2% (48.9–99.5%)	79.2% (61.4–97.1%)	0.854 (0.684–1.024)
	HC vs. 30–70%	75.3% (62.6–88%)	71.7% (52.6–90.8%)	71.4% (54.3–88.6%)	75.6% (53.9–97.3%)	75.4% (58.5–92.3%)	0.825 (0.696–0.953)
	HC vs. >70%	77.3% (64–90.6%)	66.9% (38.9–94.9%)	62.8% (38.7–86.9%)	64.5% (37.3–91.7%)	83.2% (68.5–98%)	0.856 (0.729–0.982)

LGBM: Light Gradient Boosting Machine (lightGBM), XGB: eXtreme Gradient Boosting (XGBoost).

**Table 4:** The distribution of IgG and IgM autoantibodies against unmodified, MDA, and HNE-modified peptide in oxidative model.

Attributes	HC (*n* = 30)	<30% (*n* = 30)	30–70% (*n* = 30)	>70% (*n* = 30)
IgG anti-A2M^824–841^	3.32 ± 5.35	1.51 ± 2.4	1.79 ± 2.01	1.99 ± 1.92
IgG anti-A2M^824–841^ MDA	9.87 ± 14.34	6.94 ± 11.9	4.66 ± 2.82	5.29 ± 2.8
IgG anti-ApoB100^4022–4040^	4.22 ± 7.25	3.75 ± 8.75	1.87 ± 1.19	1.78 ± 1.19
IgG anti-ApoB100^4022–4040^ MDA	1.97 ± 2.57	5.89 ± 15.61	2.21 ± 2.26	2.32 ± 3.87
IgG anti-A1AT^284–298^	3.63 ± 7.87	2.27 ± 2.75	2.39 ± 2.54	2.03 ± 1.16
IgG anti-A1AT^284–298^ MDA	4.26 ± 4.51	2.79 ± 1.79	3.42 ± 2.69	3.04 ± 1.36
IgG anti-IGKC^76–99^	2.37 ± 2.55	2.86 ± 4.22	1.74 ± 2.37	3.09 ± 5.72
IgG anti-IGKC^76–99^ MDA	4.41 ± 7.07	1.81 ± 3.02	1.52 ± 1.64	1.68 ± 1.43
IgM anti-A2M^824–841^	0.95 ± 0.53	0.6 ± 0.28	0.65 ± 0.52	0.59 ± 0.31
IgM anti-A2M^824–841^ MDA	2.03 ± 1.36	1.35 ± 0.61	1.57 ± 1.14	1.37 ± 0.65
IgM anti-ApoB100^4022–4040^	1.41 ± 0.96	1.02 ± 0.63	1.23 ± 1.53	1.18 ± 1.2
IgM anti-ApoB100^4022–4040^ MDA	1.41 ± 1.12	0.99 ± 0.43	1.18 ± 1.24	0.83 ± 0.36
IgM anti-A1AT^284–298^	1.41 ± 1.37	0.81 ± 0.7	1.8 ± 3.96	0.8 ± 1.09
IgM anti-A1AT^284–298^ MDA	1.17 ± 0.5	0.91 ± 0.37	0.93 ± 0.47	0.78 ± 0.37
IgM anti-IGKC^76–99^	3.54 ± 8.99	1.21 ± 1.06	16.3 ± 79.05	1.15 ± 1.97
IgM anti-IGKC^76–99^ MDA	0.88 ± 0.76	0.47 ± 0.26	0.54 ± 0.5	0.39 ± 0.24
IgM anti-CFAH^1211–1230^	17.54 ± 17.8	11.2 ± 9.08	13.26 ± 17.33	12.59 ± 19.43
IgG anti-CFAH^1211–1230^	26.13 ± 15.09	21.73 ± 31.63	23.53 ± 25.34	25.07 ± 25.65
IgM anti-CFAH^1211–1230^ HNE	15.71 ± 10.27	12.34 ± 8.28	12.44 ± 9.66	11.01 ± 11.05
IgG anti-CFAH^1211–1230^ HNE	26.98 ± 14.4	23.91 ± 28.53	24.71 ± 26.29	24.38 ± 20.02
IgM anti-HPT^78–108^	11.89 ± 6.34	10.95 ± 9.51	9.94 ± 7.06	8 ± 4.44
IgG anti-HPT^78–108^	37.11 ± 16.95	32.09 ± 36.49	34.67 ± 31.77	36.63 ± 26.47
IgM anti-HPT^78–108^ HNE	6.73 ± 4.14	6.9 ± 7.13	5.74 ± 5.53	4.43 ± 2.97
IgG anti-HPT^78–108^ HNE	27.67 ± 13.05	26.27 ± 27.46	26.6 ± 24.57	26.27 ± 18.45
IgG anti-IGKC^2–19^	29.36 ± 11.95	22.56 ± 19.62	26.27 ± 17.36	19.66 ± 10.1
IgM anti-IGKC^2–19^	44.88 ± 9.8	36.22 ± 11.98	33.87 ± 24.54	31.56 ± 21.59
IgM anti-IGKC^2–19^ HNE	30.11 ± 15.84	18.16 ± 15.37	19.99 ± 24.36	14.96 ± 14.68
IgG anti-IGKC^2–19^ HNE	42.7 ± 11.69	33.82 ± 37.21	34.86 ± 28.84	43.94 ± 36.92
IgM anti-THRB^328–345^	30.08 ± 26.5	16.98 ± 11.87	23.5 ± 37.37	18.83 ± 26.25
IgG anti-THRB^328–345^	88.48 ± 32.89	63.24 ± 46.2	71.84 ± 48.89	74.58 ± 46.34
IgM anti-THRB^328–345^ HNE	12.01 ± 11.93	7.96 ± 7.79	6.11 ± 5.22	7.23 ± 8.35
IgG anti-THRB^328–345^ HNE	32.16 ± 13.36	28.59 ± 35.45	30.46 ± 27.26	30.04 ± 19.74

**Table 5:** The machine learning models incorporated autoantibodies against unmodified, MDA, and HNE-modified peptides.

	Groups	Accuracy (95%CI)	Precision (95%CI)	f1 Score (95%CI)	Sensitivity (95%CI)	Specificity (95%CI)	AUC (95%CI)
LGBM	IgG anti-IGKC HNE, IgM anti-A1AT MDA, IgM anti-IGKC MDA, IgG anti-A2M MDA, IgG anti-A1AT MDA, IgM anti-CFAH HNE						
	HC vs. <30%	75.7% (62.4–88.9%)	74.8% (52.4–97.2%)	72% (53.6–90.4%)	75.1% (51.7–98.6%)	77.2% (57.5–96.9%)	0.848 (0.706–0.99)
	HC vs. 30–70%	76.1% (62.6–89.6%)	72.2% (49.3–95.1%)	71.3% (51.7–90.9%)	75.7% (51.6–99.7%)	76.6% (58.1–95.2%)	0.845 (0.687–1.002)
	HC vs. >70%	82.7% (72.3–93.1%) *	74.5% (51.5–97.5%) *	71.9% (52.2–91.5%) *	75.2% (50.7–99.6%) *	86.4% (73.8–99.1%)	0.904 (0.783–1.025) *
	**Groups**	**Accuracy (95%CI)**	**Precision (95%CI)**	**f1 Score (95%CI)**	**Sensitivity (95%CI)**	**Specificity (95%CI)**	**AUC (95%CI)**
XGB	IgG anti-IGKC HNE, IgM anti-A1AT MDA, IgM anti-IGKC MDA						
	HC vs. <30%	78.2% (64.8–91.6%)	76.8% (55.6–98%)	75% (57.8–92.2%)	78.4% (57.3–99.5%)	78.8% (59.1–98.5%)	0.847 (0.696–0.999)
	HC vs. 30–70%	78.6% (66.4–90.8%)	76.6% (55.5–97.7%)	74.2% (56.9–91.4%)	77.1% (55.4–98.8%)	79.9% (62.3–97.5%)	0.881 (0.751–1.011)
	HC vs. >70%	86.1% (76.2–96%) *	81.7% (60.5–103%) *	77.2% (59–95.5%) *	78.6% (55.8–101.4%) *	90.4% (79.6–101.1%) *	0.935 (0.846–1.024) *

* means *p* < 0.05; LGBM: Light Gradient Boosting Machine (lightGBM), XGB: eXtreme Gradient Boosting (XGBoost).

## Data Availability

Not applicable.

## Supplementary Materials

The following supporting information can be downloaded at: https://www.mdpi.com/article/10.3390/diagnostics12102269/s1, Supplementary Figure S1: Dot plots of plasma concentration of autoantibodies: IgG anti-BSA and IgG anti-HNE modified BSA (A), IgM anti-BSA and IgM anti-HNE-modified BSA (B) in healthy controls (HCs), rheumatoid arthritis (RA) patients and RA patients with coronary artery disease (CAD); Supplementary Figure S2: Dot plots of plasma concentration of autoantibodies: IgG anti-CFAH^1211–1230^ and IgG anti-CFAH^1211–1230^ HNE (A), IgG anti-HPT^78–108^ and IgG anti-HPT^78–108^ HNE (B), IgG anti-IGKC^2–19^ and IgG anti-IGKC^2–19^ HNE (C), IgG anti-THRB^328–345^ and IgG anti-THRB^328–345^ HNE (D), IgM anti-CFAH^1211–1230^ and IgM anti-CFAH^1211–1230^ HNE (E), IgM anti-HPT^78–108^ and IgM anti-HPT^78–108^ HNE (F), IgM anti-IGKC^2–19^ and IgM anti-IGKC^2–19^ HNE (G), IgM anti-THRB^328–345^ and IgM anti-THRB^328–345^ HNE (H) in healthy controls (HCs) and coronary artery disease patients with stenosis rates of <30%, 30–70%, and >70%.
