# Supplementary material for: Autoantibodies to Oxidatively Modified Peptide: Potential Clinical Application in Coronary Artery Disease
Source: Diagnostics (Basel). 2022 Sep 20;12(10):2269. doi: 10.3390/diagnostics12102269 (PMC9600024; doi:10.3390/diagnostics12102269)
Supplement: Supplementary file 1 [file diagnostics-12-02269-s001.zip › diagnostics-1880137-supplementary/Supplementary Figure S1 BSA dot plots .pptx]

## Slide 1
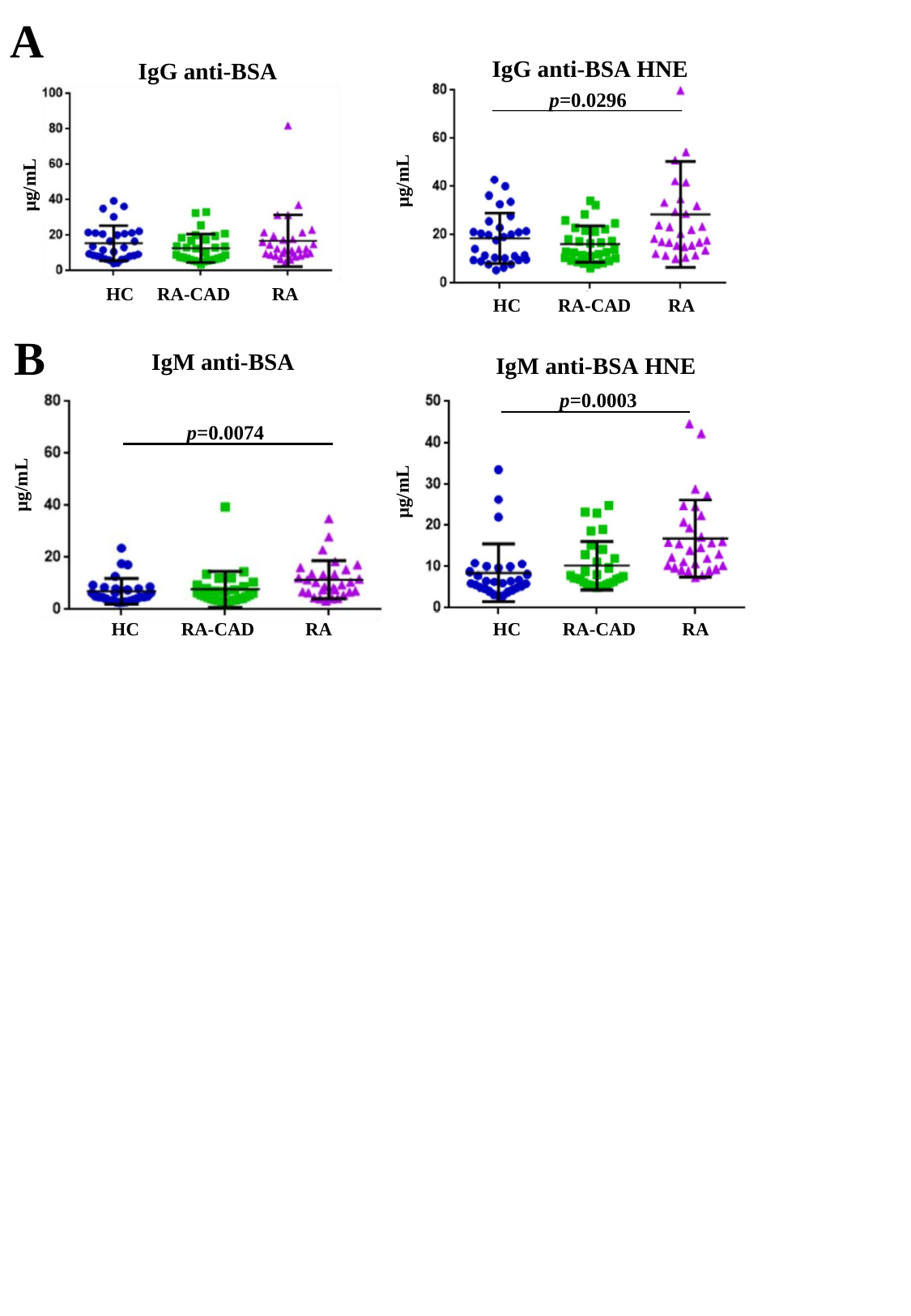

A
IgG anti-BSA HNE
p=0.0296
μg/mL
HC RA-CAD RA
IgG anti-BSA
μg/mL
HC RA-CAD RA
B
IgM anti-BSA
p=0.0074
μg/mL
HC RA-CAD RA
IgM anti-BSA HNE
p=0.0003
μg/mL
HC RA-CAD RA
