# Supplementary figures and images for: Autoantibodies to Oxidatively Modified Peptide: Potential Clinical Application in Coronary Artery Disease
Source: Diagnostics (Basel). 2022 Sep 20;12(10):2269. doi: 10.3390/diagnostics12102269 (PMC9600024; doi:10.3390/diagnostics12102269)

## Slide 1
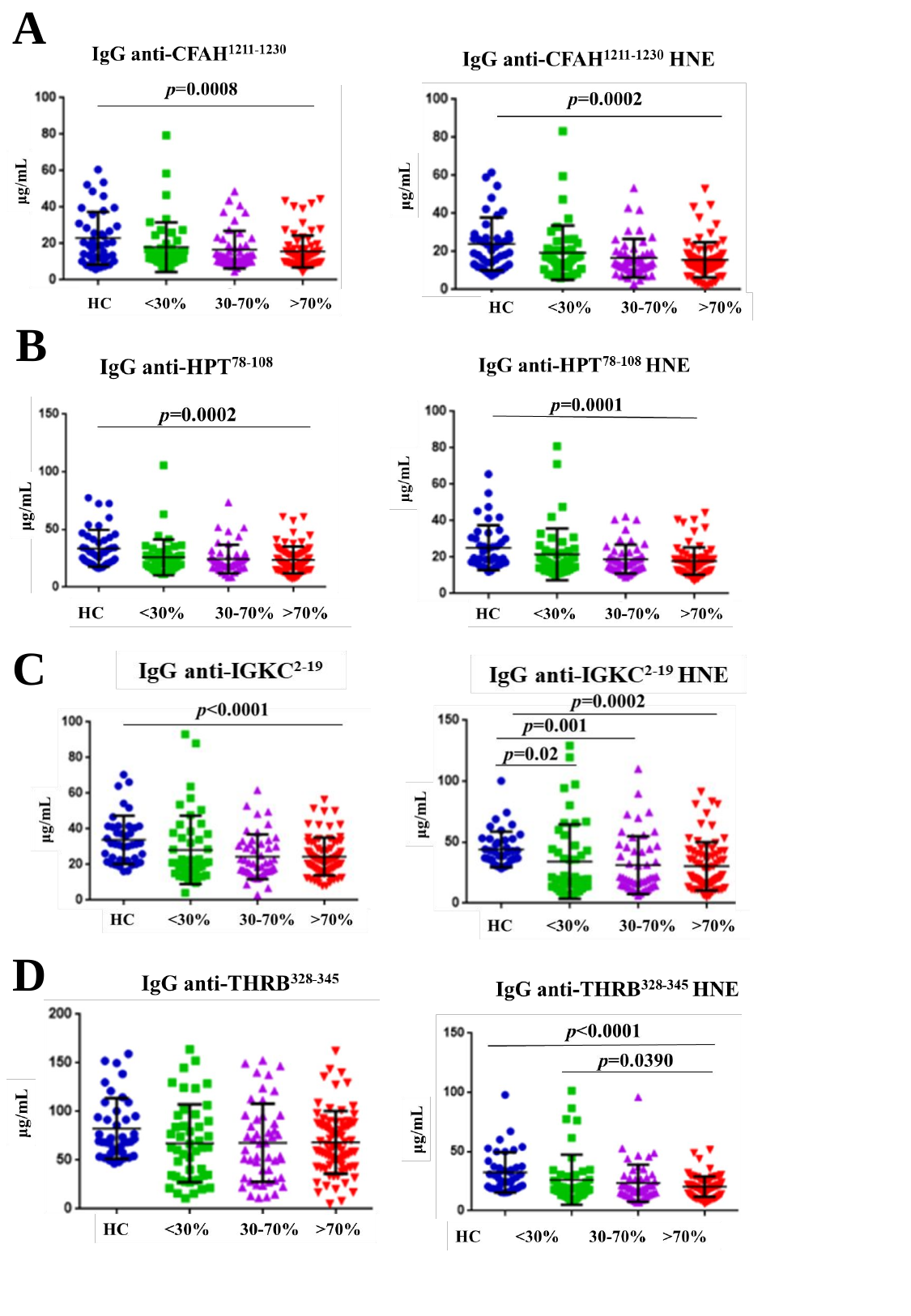

A
B
C
D

## Slide 2
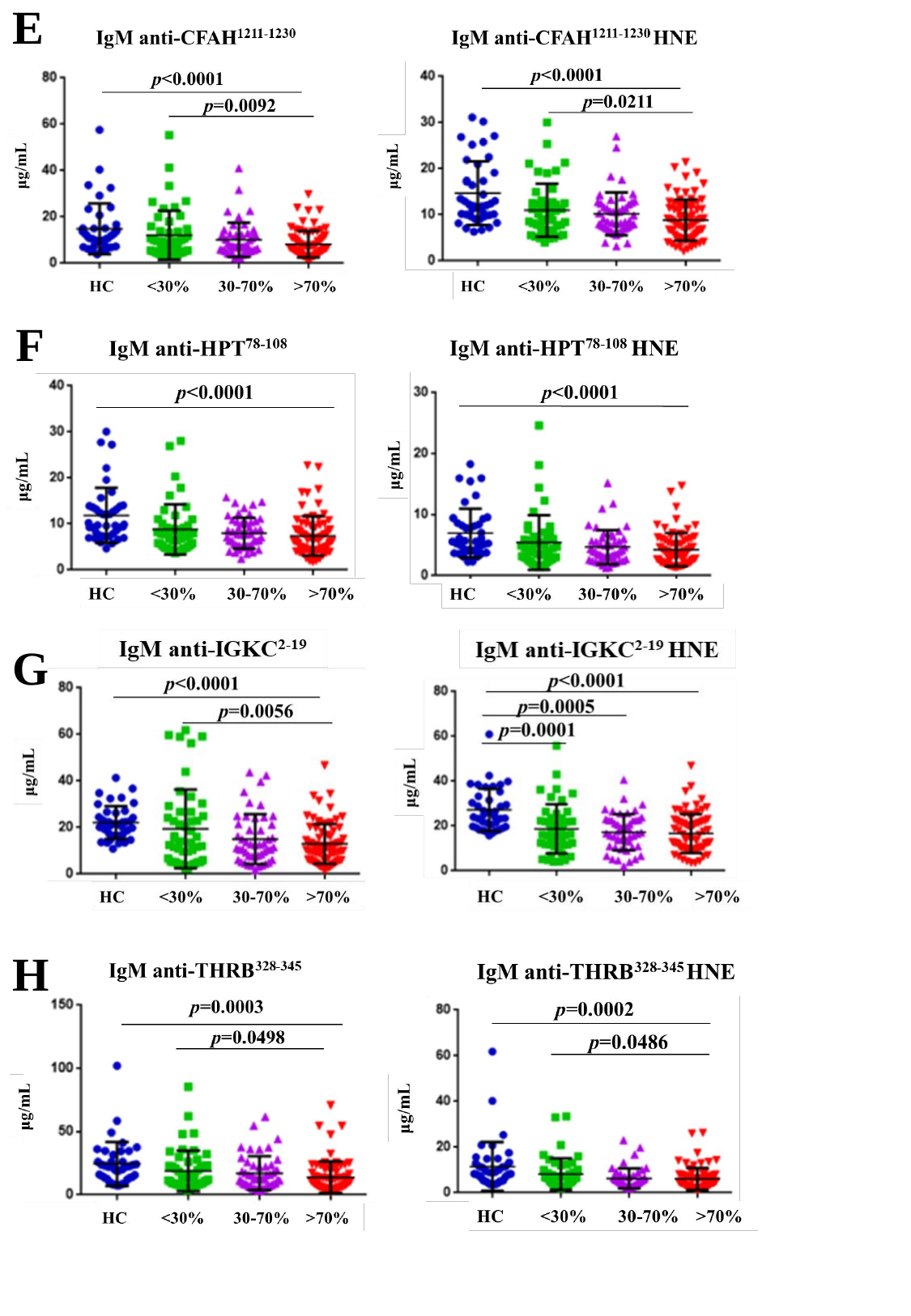

E
F
G
H

Supplement: Supplementary file 1 [file diagnostics-12-02269-s001.zip › diagnostics-1880137-supplementary/Supplementary Figure S2 Dot plots.pptx]
